# Identification and Prediction of Differentially Expressed MicroRNAs Associated with Detoxification Pathways in Larvae of *Spodoptera frugiperda*

**DOI:** 10.3390/genes15081021

**Published:** 2024-08-03

**Authors:** Yan-Ping Wang, Xing-Yu Chen, De-Qiang Pu, Chun-Yan Yi, Chang-Hua Liu, Cui-Cui Zhang, Zhen-Zhen Wei, Jing-Wei Guo, Wen-Juan Yu, Song Chen, Hong-Ling Liu

**Affiliations:** 1 Key Laboratory of Integrated Pest Management of Southwest Crops, Institute of Plant Protection, Sichuan Academy of Agricultural Sciences, Chengdu 610066, China; singular2023@outlook.com (Y.-P.W.); pdqpudeqiang@163.com (D.-Q.P.); yichunyan2023@outlook.com (C.-Y.Y.); liuchanghua2023@outlook.com (C.-H.L.); zhangcuicui2023@outlook.com (C.-C.Z.); zhenzhen-wei@hotmail.com (Z.-Z.W.); jingwei.guo66@hotmail.com (J.-W.G.); wjyu0906@163.com (W.-J.Y.); chensong523@126.com (S.C.); 2 Science and Technology Security Center, Sichuan Academy of Agricultural Sciences, Chengdu 610066, China; chenxingyu2023@outlook.com

**Keywords:** detoxification, larva, microRNAs, resistance, *S. frugiperda*, the fall armyworm

## Abstract

*Spodoptera frugiperda* poses a severe threat to crops, causing substantial economic losses. The increased use of chemical pesticides has led to resistance in *S. frugiperda* populations. Micro ribonucleic acids (MicroRNAs or miRNAs) are pivotal in insect growth and development. This study aims to identify miRNAs across different developmental stages of *S. frugiperda* to explore differential expression and predict target gene functions. High-throughput sequencing of miRNAs was conducted on eggs, 3rd instar larvae, pupae, and adults. Bioinformatics analyses identified differentially expressed miRNAs specifically in larvae, with candidate miRNAs screened to predict target genes, particularly those involved in detoxification pathways. A total of 184 known miRNAs and 209 novel miRNAs were identified across stages. Comparative analysis revealed 54, 15, and 18 miRNAs differentially expressed in larvae, compared to egg, pupa, and adult stages, respectively. Eight miRNAs showed significant differential expression across stages, validated by quantitative reverse transcription PCR (qRT-PCR). Gene Ontology and Kyoto Encyclopedia of Genes and Genomes enrichment analyses predicted target genes’ functions, identifying eight differentially expressed miRNAs targeting 10 gene families associated with detoxification metabolism, including P450s, glutathione S-transferase (GSTs), ATP-binding cassette (ABC) transporters, and sodium channels. These findings elucidate the species-specific miRNA profiles and regulatory mechanisms of detoxification-related genes in *S. frugiperda* larvae, offering insights and strategies for effectively managing this pest.

## 1. Introduction

MicroRNAs (miRNAs) are endogenous, single-stranded, non-protein-coding small RNAs approximately 19 to 24 nucleotides in length [1]. These miRNAs are ubiquitously present in animals, plants, and microorganisms, playing a crucial role in the post-transcriptional regulation of genes [2,3,4,5,6,7,8]. Studies have demonstrated that miRNAs are integral to various biological processes, including tissue growth, germ cell development, hormone action, and the development and function of the central nervous system, primarily through gene regulation [9,10]. It is estimated that miRNAs regulate the expression of more than 50% of protein-coding genes in animals [11].

Research on insect miRNAs has garnered increasing attention, leading to the discovery of numerous miRNAs across different insect species. These miRNAs regulate a wide range of physiological functions throughout insect development, such as molting, metamorphosis, oogenesis, embryogenesis, behavior, and host–pathogen interactions [12,13,14,15,16,17,18]. For instance, Dicer-1, a key gene in miRNA processing, when interfered with RNA, was found to partially inhibit the metamorphosis of *Blattella germanica* (L.) (Blattaria: Blattidae) after molting [4]. The miR-2 family influences insect metamorphosis and development via juvenile hormones [19,20,21], while miR-14 regulates insect molting by participating in the regulation of ecdysone receptors [22,23]. Additionally, miRNAs play vital roles in the growth and development of other insects such as *Bactrocera dorsalis* Hendel (Diptera: Tephritidae), *Nilaparvata lugens* (Hemiptera: Delphacidae), and *Spodoptera exigua* (Lepidoptera: Noctuidae) [24,25,26].

Given their significant role in post-transcriptional gene regulation, binding to specific sequences of target mRNA, miRNA can degrade target mRNA or inhibit its normal translation, thereby regulating the expression of target genes. miRNAs are also implicated in the molecular mechanisms that enhance drug resistance by regulating detoxification genes in insects [27,28]. Detoxification metabolic resistance is crucial for pest resistance development. For instance, miR-7a and miR-8519 could upregulate the expression of the ryanodine receptor (RyR), enhancing resistance to chlorantraniliprole [29]. miR-998-3p can negatively regulate the expression of ABCC2, contributing to the detoxification of BtCry1Ac toxins in three typical lepidopteran pests: *Helicoverpa armigera* (Lepidoptera: Noctuidae), *S. exigua*, and *Plutella xylostella* (Lepidoptera: Plutellidae) [30]. Furthermore, miRNAs novel-85 and novel-191 can target CYP6ER1 and carboxylesterase 1 (CarE1), significantly altering *N. lugens*’ susceptibility to nitenpyram [31]. In addition, miR-4133-3p could regulate the detoxification of gossypol and tannic acid by targeting the cytochrome P450 4CJ1 (CYP4CJ1) in Aphis gossypii Glover (Hemiptera: Aphididae) [32].

The fall armyworm, *S. frugiperda* Smith, 1797 (Lepidoptera: Noctuidae), is a major migratory agricultural pest of global concern [33]. This omnivorous pest has a strong migratory ability, a high reproductive rate, and a short life cycle, damaging 353 species of plants across 76 families, including major crops such as corn, sorghum, sugarcane, barley, rice, pepper, wild oat, and potato [34,35]. The primary method of controlling this pest relies on chemical pesticides, which have proven to be effective [36]. However, reports indicate that *S. frugiperda* has developed resistance to various insecticides, including bromides, neonicotinoids, and pyrethroids [37,38,39]. Insect resistance to insecticides is mainly attributed to enhanced metabolism and alterations in target site structures [40,41,42].

To date, some miRNAs have been identified in *S. frugiperda*, with functional studies primarily focusing on antiviral immune defense and adaptive evolution [43,44]. However, systematic identification and functional analysis of miRNAs related to detoxification metabolism in *S. frugiperda* remain limited [45]. Using high-throughput sequencing of miRNA libraries, this study investigated miRNAs in *S. frugiperda* at different developmental stages, analyzed differentially expressed miRNAs in larvae, and identified target genes and functional predictions of these miRNAs across four stages. This research aims to elucidate the regulatory mechanisms of *S. frugiperda* growth and development, analyze detoxification-related target genes, and provide new strategies for the effective management of *S. frugiperda* outbreaks and damage.

## 2. Materials and Methods

### 2.1. Insect Collection and Rearing

The larvae of *S. frugiperda* were collected from a cornfield at the Xindu Base of the Sichuan Academy of Agricultural Sciences, China. They were continuously raised in the insect laboratory of Plant Conservation Institute of Sichuan Academy of Agricultural Sciences. The larvae were reared continuously in a light incubator under controlled conditions (16/8 hr light/dark (L/D) cycle, 65% humidity, 25 °C) and fed with an artificial diet [46], which consisted of the following components: wheat germ powder 280 g, soy protein powder 90 g, yeast powder 35 g, agar 25 g, vitamin B complex 0.2 g, cholesterol 12 g, sorbate 2 g, ascorbic acid 12 g, methyl p-hydroxybenzoate 5 g, formaldehyde 4 mL, penicillin 0.2 g, and distilled water 1500 mL. Sampling periods at different developmental stages were as follows: 500 eggs were collected within 24 h post-laying, 20–30 third instar larvae were collected within 12 h post-molting, 3–5 pupae were collected within 36 h post-pupation, and 2–3 adults were collected within 12 h post-emergence. Each sample contained 0.3–0.5 g and had three biological replicates. Samples were placed in 2.0 mL RNase-free centrifuge tubes with TRIzol reagent (Invitrogen Life Technologies, Carlsbad, CA, USA) for temporary storage. After collection, samples were stored in an ultra-low temperature freezer at −80 °C.

### 2.2. Small RNA Library Preparation and Sequencing

Total RNA was extracted using TRIzol reagent (Invitrogen Life Technologies, Carlsbad, CA, USA) following the manufacturer’s instructions. RNA concentrations were measured using a NanoDrop spectrophotometer (Thermo Scientific, Sunnyvale, CA, USA), and RNA quality and integrity were assessed with an Agilent 2100 Bioanalyzer (Agilent Technologies, Waldbronn, Germany). Total RNA was used to construct small RNA (sRNA) libraries with the NEBNext Multiplex Small RNA Library Prep Set for Illumina (New England Biolabs, Ipswich, MA, USA).

One microgram of total RNA from *S. frugiperda* samples was ligated to 3′ and 5′ adapters using Ligation Enzyme Mix. The samples were then reverse-transcribed using Superscript II reverse transcriptase. The sRNA libraries were subjected to quality control, and the average insert size was approximately 140 to 150 bp. cDNA was prepared using random primers and a cDNA Synthesis Kit (Invitrogen, CA, USA). The sequencing library was quantified using the Agilent High Sensitivity DNA assay on an Agilent Bioanalyzer (Agilent Technologies, Germany) and sequenced on a NovaSeq 6000 (Illumina) in PE150 mode at Personal Biotechnology Co., Ltd. (Shanghai, China).

### 2.3. Unigene Assembly, Analysis, and Annotation

Raw sequencing reads underwent quality control using FastQC program (Babraham Bioinformatics, Cambridge, UK) [47], and then the raw data were filtered using the Personalbio company’s self-developed script. Clean reads ranging from 18 to 36 nt were filtered, and deduplication was performed to obtain unique reads for subsequent analysis. The reference genome index was built using Bowtie2 v2.5.1 [48], and de-duplicated clean reads were mapped to the reference genome using miRDeep2 v2.0 (https://sourceforge.net/projects/mireap/, accessed on 1 October 2023) [49]. Unique reads were aligned to known miRNAs in the miRBase R.22. database (http://www.mirbase.org/, accessed on 1 October 2023) and annotated with other non-coding RNAs. Sequences without annotations were analyzed using mireap v0.2 for novel miRNA prediction. Annotation results were organized according to the priority: known miRNA > piRNA > rRNA > tRNA > snRNA > snoRNA > novel miRNA, ensuring each small RNA had a unique annotation.

### 2.4. Differential Expression Analysis of miRNAs

The reads count of miRNAs was determined based on sequences aligned to mature miRNAs. The highest abundance among miRNAs of the same name was used for subsequent analysis. Differentially expressed miRNAs were identified using DESeq v1.39.0 [50], with transcripts showing a log2 fold change > 1 and *p*-value < 0.05 considered significant. Bidirectional cluster analysis of all miRNAs and samples was performed using the R package Pheatmap v1.0.12 [51], with the Euclidean method for distance calculation and Complete Linkage for hierarchical clustering.

### 2.5. Quantitative Reverse Transcription PCR

Eight differentially expressed miRNAs were selected for qRT-PCR using initial RNA samples. Stem-loop RT primers and gene-specific primers were designed (Table 1). Reverse transcription was performed with the PrimeScript RT reagent kit with gDNA Eraser (Takara, Tokyo, Japan), the reference gene Actin reverse transcription was performed using the general primers of the kit, and the specific primers of miRNAs were reversed. We used 1 μg of total RNA under the following conditions: 16 °C for 30 min, 42 °C for 30 min, and 85 °C for 5 min. The reference gene qRT-PCR reaction contained 2 μL of diluted cDNA (15 ng), 10 μL of 2× AceQ qPCR SYBR Green Master mix (Vazyme, Nanjing, China), and 10 μM of each primer in a 20 μL total volume [52]. The miRNAs qRT-PCR reaction contained 2 μL of diluted cDNA (15 ng), 10 μL of 2× AceQ Universal U^+^ Probe Master mix (Vazyme, China), 10 μM probe, and 10 μM of each primer in a 20 μL total volume [53]. The conditions used were: 95 °C for 5 min, followed by 40 cycles of 95 °C for 10 s, 60 °C for 30 s, and a melting curve from 68 °C to 95 °C. The Applied Biosystems QuantStudio 6 Flex system (Thermo Scientific, Sunnyvale, CA, USA) was used with Sf-actin as an endogenous control. The 2^−ΔΔCT^ method was used to calculate the relative expression levels of miRNAs [54]. The qRT-PCR included three technical and biological replicates.

### 2.6. Gene Ontology (GO) and Kyoto Encyclopedia of Genes and Genomes (KEGG) Enrichment Analysis

Target genes of differentially expressed miRNAs were predicted using miRanda v3.3a (https://bioweb.pasteur.fr/packages/pack@miRanda@3.3a/, accessed on 13 December 2023), considering the 3′ untranslated region (UTR) sequences of *S. frugiperda* mRNAs. GO (http://geneontology.org/, accessed on 13 December 2023) and KEGG (http://www.kegg.jp/, accessed on 13 December 2023) enrichment analyses were performed on the predicted target genes. GO enrichment was conducted using topGO v2.50.0 [55], with significant enrichment determined by *p*-value < 0.05 using the hypergeometric distribution method. KEGG pathway enrichment analysis was performed using clusterProfiler v4.6.0 [56], focusing on pathways with *p*-values < 0.05.

### 2.7. Statistical and Data Analysis

Statistical analyses were conducted using Statistical Product and Service Solutions (SPSS) v20.0 (SPSS Inc., Chicago, IL, USA). Comparisons were performed using Student’s *t*-test, with a *p*-value < 0.05 considered statistically significant.

## 3. Results

### 3.1. Small RNA Sequencing Data in S. frugiperda

High-throughput sequencing generated twelve sRNA libraries for *S. frugiperda* at four different developmental stages: egg, larva, pupa, and adult. The transcriptome data for these stages contain 20,128,612, 16,315,920, 23,520,239, and 17,593,809 raw reads, respectively (Table 2). The total raw sequences for the four developmental stages exceeded 10 Mb, with high-quality reads constituting 91–97%, indicating good sequencing quality. After filtering clean reads (≥18 nt), the number of valid sequences obtained for egg, larva, pupa and adult stages were 17,231,905, 3,731,980, 17,141,575, and 12,107,130 reads, respectively.

**Figure 1:**
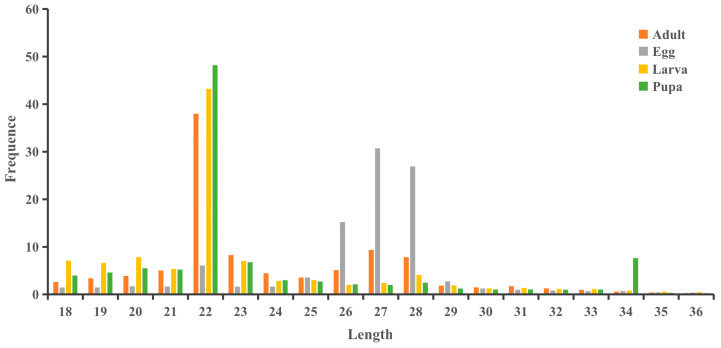
Length distribution of sRNAs in different stages of *S. frugiperda*. X-axis indicates sequence length, while Y-axis shows the frequence in percentage.

The sRNA lengths for the four developmental stages of *S. frugiperda* ranged from 18 to 36 nt (Figure 1). In larvae, pupae, and adults, the 22 nt sequences were the most abundant, accounting for 43.20%, 48.18%, and 38.99% of the total, respectively. In eggs, the 27 nt sequences were the most abundant, making up 30.72% of the total.

### 3.2. Small RNA Classification and Annotation

**Figure 2:**
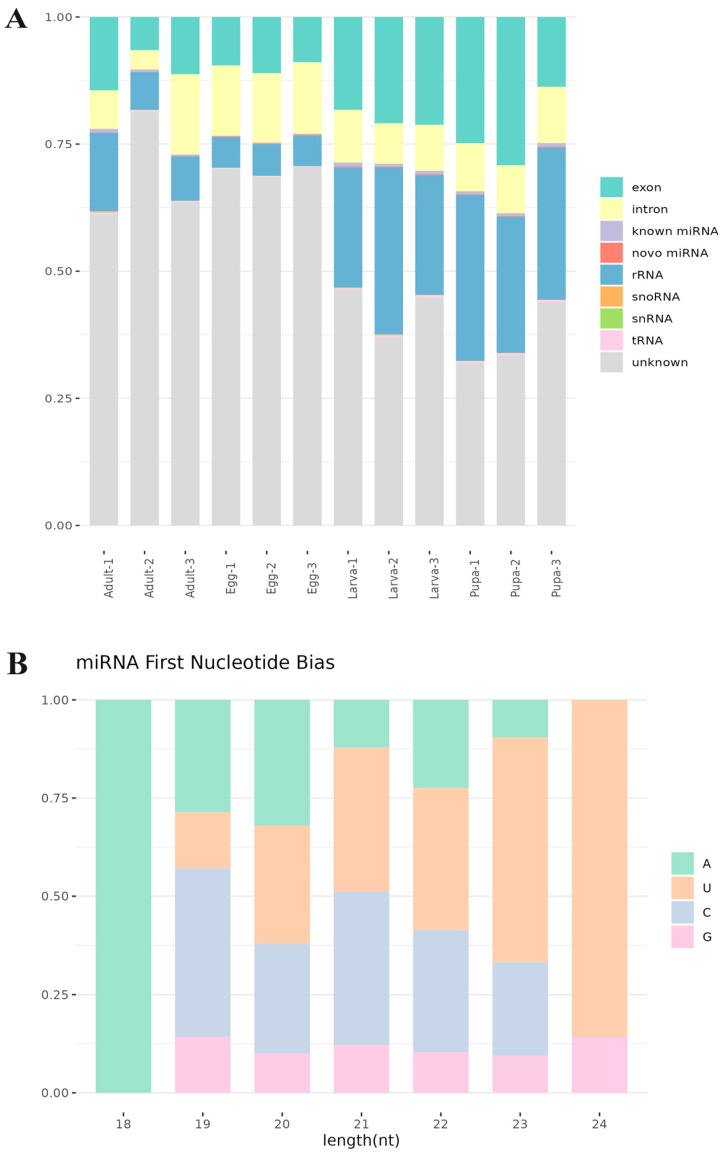

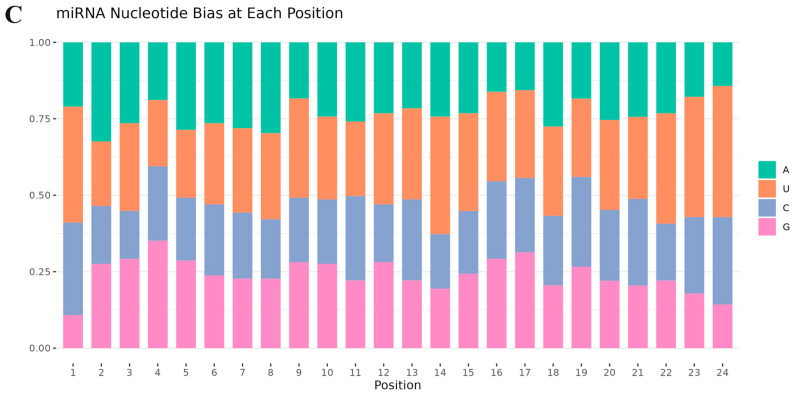
Composition of sRNAs in *S. frugiperda*. (**A**) Annotation of sRNAs. X-axis shows the sample number, whereas Y-axis shows the proportion of the de-duplicated sequences annotated to each sRNA to the total de-duplicated sequences. (**B**) First base bias of the miRNAs in *S. frugiperda*. X-axis shows the miRNAs length, and Y-axis shows the frequency in percentage. (**C**) Base bias of the miRNAs at each position. X-axis shows the nucleotide position, and Y-axis shows the frequency in percentage.

To identify known non-coding RNAs, the precursor and mature sequences of miRNAs for the species were downloaded from miRBase. The deduplicated sequences were aligned to these miRNAs to annotate sRNAs at different developmental stages of *S. frugiperda* (Figure 2A, Table S1). Among the annotated sRNAs, ribosomal RNA (rRNA) was the most abundant, constituting 32.89% of the total. Larvae and pupae had higher rRNA quantities than adults and eggs. tRNA, snoRNA, and snRNA were significant components of sRNAs, with maximum proportions of 0.40%, 0.12%, and 0.14%, respectively. A significant proportion of sRNAs (31.94–81.26%) remained unannotated across all developmental stages.

The nucleotide preference at the first base of miRNAs in different developmental stages of *S. frugiperda* was analyzed (Figure 2B), with most sRNAs showing a preference for U (uracil) at the first base. In all samples, the first base of 18 bp sRNAs preferred A (adenine). Different nucleotide positions exhibited different preferences (Figure 2C). The preference for U at the 5′ end is a conserved characteristic of miRNAs.

### 3.3. Identification of Known and Novel miRNAs in S. frugiperda

**Figure 3:**
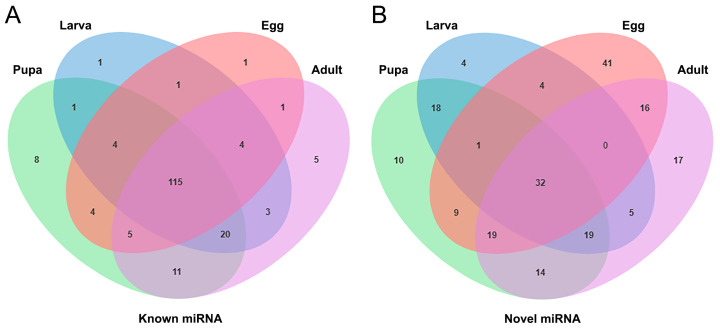
Venn diagram of differential expression in known (**A**) and novel (**B**) miRNAs of *S. frugiperda*. Numbers indicate shared miRNAs.

Precursor and mature sequences of miRNAs were downloaded from miRBase, and deduplicated sequences were aligned to these miRNAs for annotation. A total of 135, 149, 168, and 164 known miRNAs were identified in eggs, larvae, pupae, and adults, respectively. Among these, 115 known miRNAs were present across all four developmental stages (Figure 3A). Additionally, novel miRNAs were predicted from sequences not annotated with any information using mireap analysis, resulting in 147, 83, 122, and 138 novel miRNAs identified in eggs, larvae, pupae, and adults, respectively. Among these, 32 novel miRNAs were common across all four stages (Figure 3B). The ten most highly expressed miRNAs were detected (Table S2), with sfr-miR-2766-3p, sfr-miR-279a-3p, and sfr-miR-10-5p being the most abundantly expressed.

### 3.4. Identification of Differentially Expressed miRNAs in Larvae

miRNAs expressed in both larvae and other developmental stages (eggs, pupae, and adults) were selected based on differential expression (log2 fold change > 1) and significance (*p*-value < 0.05). The results showed 67 differentially expressed miRNAs across the four developmental stages. The number of differentially expressed miRNAs between larvae and eggs, pupae, and adults were 54, 15, and 18, respectively (Table 3).

**Figure 4:**
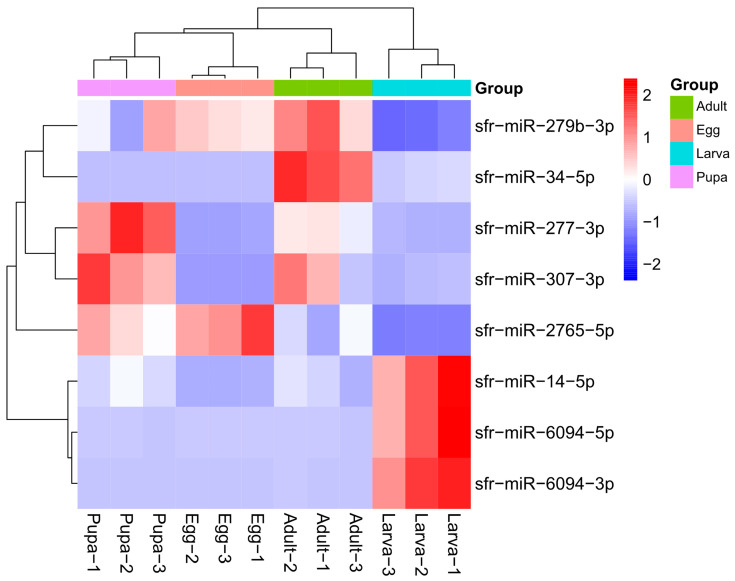
Heatmap of differentially expressed miRNAs in different stages of *S. frugiperda*. Red indicates upregulation; blue means downregulation.

Eight miRNAs showed differential expression in *S. frugiperda* larvae compared to the other three stages (Figure 4). Among them, sfr-miR-14-5p, sfr-miR-6094-5p, and sfr-miR-6094-3p were significantly upregulated in larvae compared to the other stages. The most significantly upregulated miRNA in larvae was sfr-miR-6094-3p, with expression levels increasing by 223.41, 482.67, and 79.76 times compared to eggs, pupae, and adults, respectively. sfr-miR-6094-5p and sfr-miR-14-5p were also highly upregulated, with sfr-miR-6094-5p showing increases of 209.54, 253.92, and 87.57 times, and sfr-miR-14-5p showing increases of 23.89, 3.69, and 5.21 times, compared to eggs, pupae, and adults, respectively.

Conversely, the most significantly downregulated miRNA in larvae was sfr-miR-2765-5p, with expression decreasing by 74.20, 61.11, and 37.06 times compared to eggs, pupae, and adults, respectively. sfr-miR-279b-3p was also significantly downregulated, with decreases of 3.89, 3.77, and 6.97 times compared to eggs, pupae, and adults, respectively. Additionally, sfr-miR-277-3p and sfr-miR-307-3p were downregulated in larvae compared to pupae and adults, and sfr-miR-34-5p was downregulated in larvae compared to adults.

### 3.5. qRT-PCR Validation of Differentially Expressed miRNAs in Larvae

**Figure 5:**
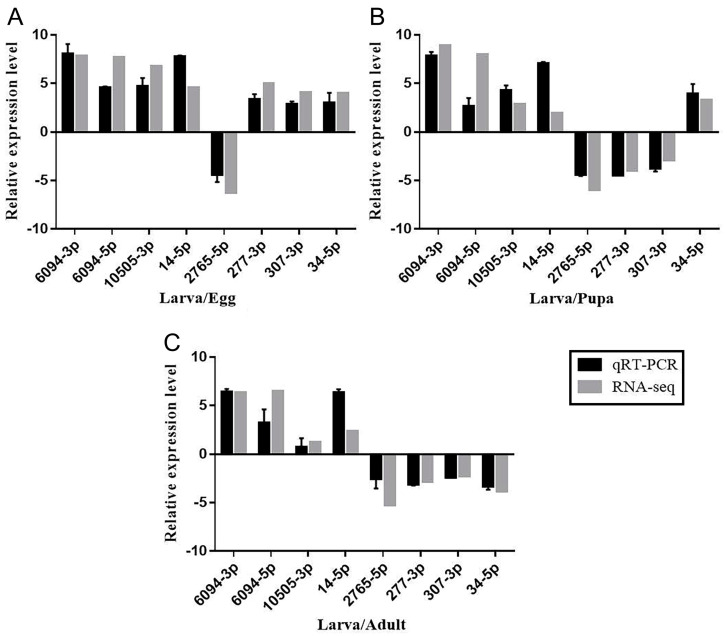
Relative expression level of selected differentially expressed miRNAs of *S. frugiperda* between qRT-PCR and RNA-seq in (**A**) larvae vs. eggs, (**B**) larvae vs. pupae, and (**C**) larvae vs. adults.

To further confirm the miRNA sequencing results, eight miRNAs (miR-6094-5p, miR-6094-3p, miR-14-5p, miR-10505-3p, miR-2765-5p, miR-277-3p, miR-307-3p, and miR-34-5p) were randomly selected for qRT-PCR based on their differential expression in larvae compared to the other three developmental stages. The qRT-PCR results showed consistent expression patterns with RNA-seq. Compared with the egg stage, the expression of miR-2765-5p was downregulated, and the expression of other miRNAs was upregulated (Figure 5A). Compared with the pupa stage, the expressions of miR-6094-5p, miR-6094-3p, miR-10505-3p, miR-14-5p, and miR-34-5p were upregulated, while the expressions of miR-2765-5p, miR-277-3p, and miR-307-3p were downregulated (Figure 5B). Compared with the adult stage, the expressions of miR-6094-5p, miR-6094-3p, and miR-14-5p were upregulated, while the expressions of miR-2765-5p, miR-277-3p, miR-307-3p, and miR-34-5p were downregulated (Figure 5C). This suggests that the orientation of regulation and expression pattern of these miRNAs validated by RT-qPCR was consistent with the results from the miRNA sequencing, thus confirming the reliability and repeatability of the miRNA sequencing method.

### 3.6. Prediction of Targeted Genes

Using miRanda, the 3′ UTR sequences of the species’ mRNAs were targeted for the prediction of differentially expressed miRNA target genes. A total of 2019 target genes and 66,004 target sites were predicted for the 67 differentially expressed miRNAs in *S. frugiperda* larvae.

**Figure 6:**
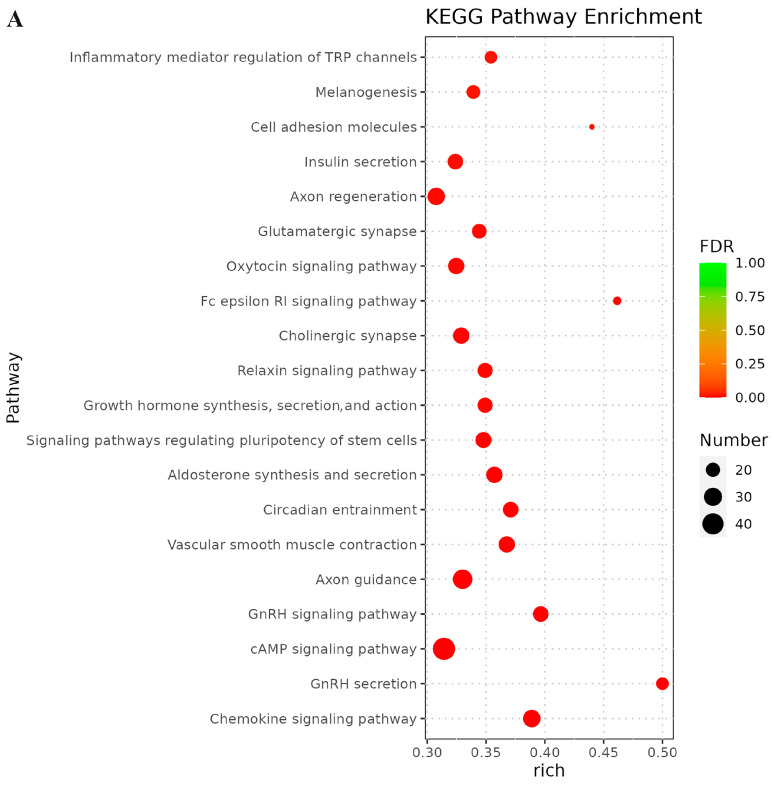

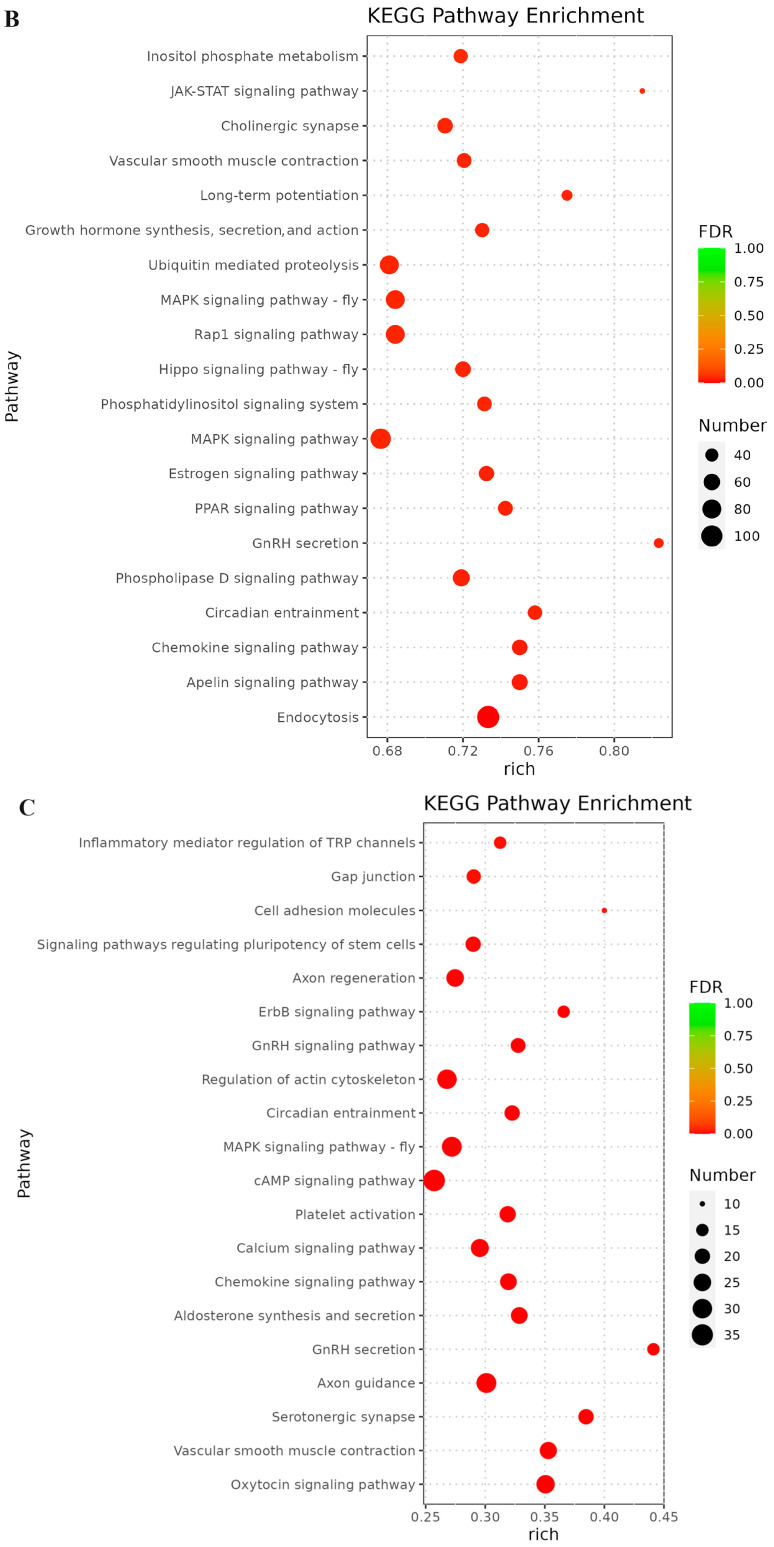
The top 20 significantly enriched KEGG pathways of *S. frugiperda* in (**A**) larvae vs. adults, (**B**) larvae vs. eggs, and (**C**) larvae vs. pupae. X-axis shows the enrichment factor, and Y-axis shows the pathway names. Definitions of the pathways are available at https://www.kegg.jp/kegg/pathway.html. The enrichment degree was measured by Rich factor value and the number of miRNA target genes enriched on this pathway. Rich factor refers to the ratio of the number of differential miRNA target genes enriched in this pathway to the number of differential miRNA target genes annotated. The greater the Rich factor, the greater the degree of enrichment.

To further investigate the functions of miRNAs in the *S. frugiperda* transcriptome, the top twenty significantly enriched KEGG pathways were analyzed. In larvae vs. adults (Figure 6A), the most significantly enriched pathways were chemokine signaling pathway, gonadotropin-releasing hormone (GnRH) secretion, cyclic adenosine monophosphate (cAMP) signaling pathway, GnRH signaling pathway, and axon guidance. In larvae vs. eggs (Figure 6B), the top pathways were endocytosis, apelin signaling pathway, chemokine signaling pathway, circadian entrainment, and phospholipase D signaling pathway. In larvae vs. pupae (Figure 6C), the most significantly enriched pathways were oxytocin signaling pathway, vascular smooth muscle contraction, serotonergic synapse, axon guidance, and GnRH secretion.

**Figure 7:**
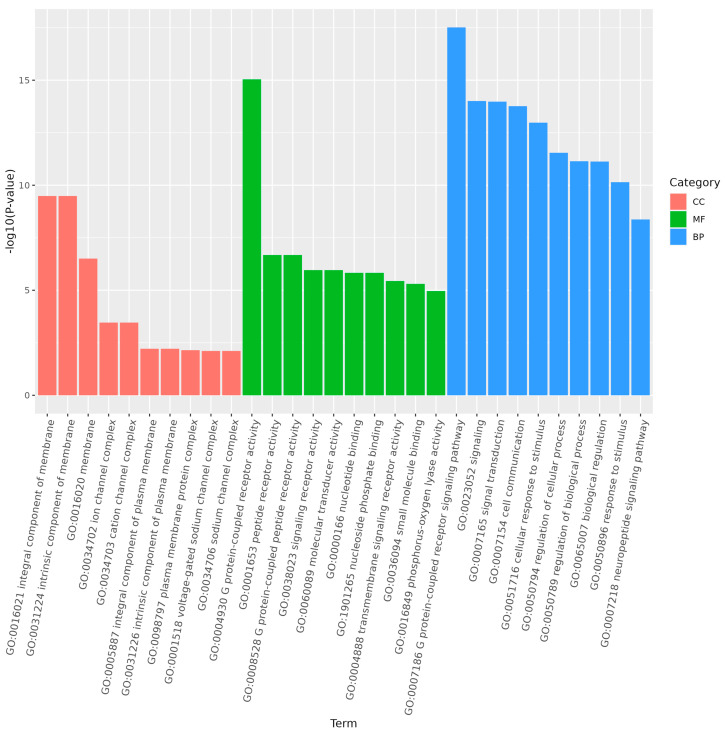
The top 30 GO terms of the target genes of differentially expressed miRNAs of *S. frugiperda* in larvae vs. adults. The enrichment degree was measured using the false discovery rate (FDR) value and the number of miRNA target genes enriched on this pathway. FDR generally ranges from 0 to 1, and the closer it is to zero, the more significant the enrichment. The top 30 pathways with the smallest FDR value, that is, the most significant enrichment, were selected for display.

**Figure 8:**
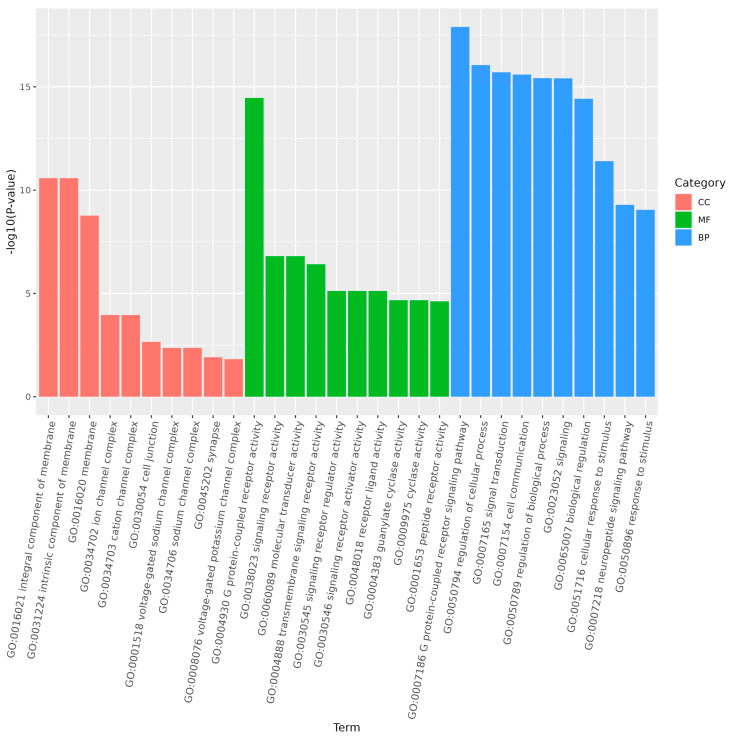
The top 30 GO terms of the target genes of differentially expressed miRNAs of *S. frugiperda* in larvae vs. pupae. The enrichment degree was measured by FDR value and the number of miRNA target genes enriched on this pathway. FDR generally ranges from 0 to 1, and the closer it is to zero, the more significant the enrichment. The top 30 pathways with the smallest FDR value, that is, the most significant enrichment, were selected for display.

**Figure 9:**
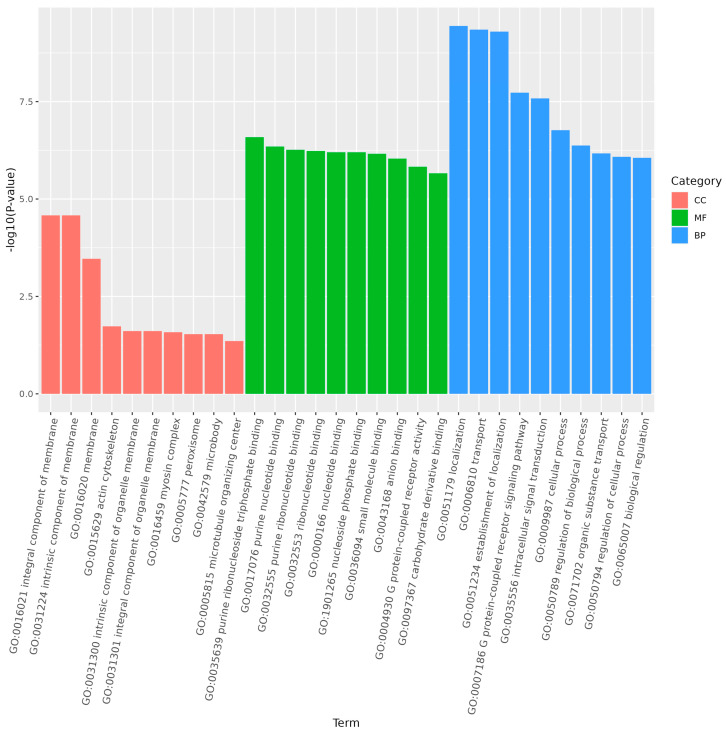
The top 30 GO terms of the target genes of differentially expressed miRNAs of *S. frugiperda* in larvae vs. eggs. X-axis shows GO terms, and Y-axis shows −log10 (*p*-value). The enrichment degree was measured by FDR value and the number of miRNA target genes enriched on this pathway. FDR generally ranges from 0 to 1, and the closer it is to zero, the more significant the enrichment. The top 30 pathways with the smallest FDR value, that is, the most significant enrichment, were selected for display.

GO enrichment analysis of the target genes of differentially expressed miRNAs showed that the functions of differentially expressed genes in larvae compared to the other three stages were mainly distributed across cellular components, molecular functions, and biological processes. In larvae, adults, and pupae, the G protein-coupled receptor signaling pathway was a significantly enriched subgroup (Figure 7 and Figure 8). In larvae and eggs, localization was a significantly enriched subgroup (Figure 9).

**Figure 10:**
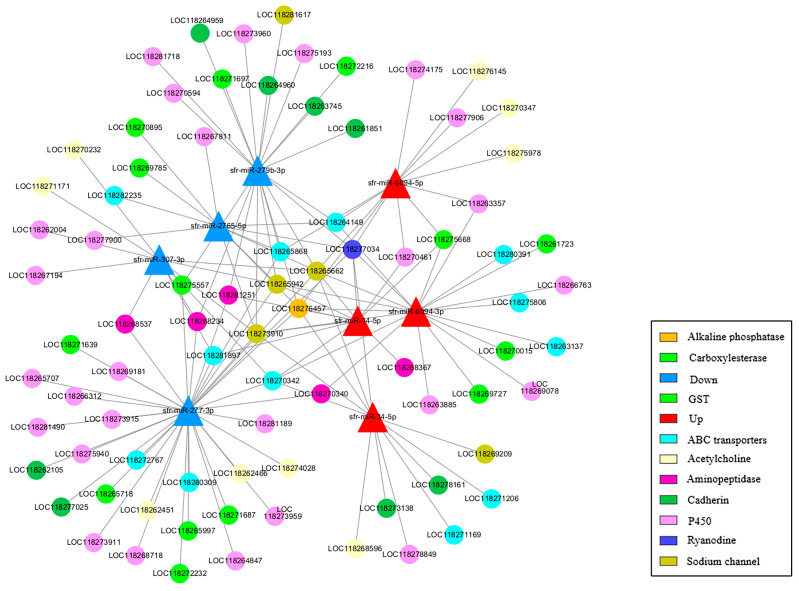
A miRNA–mRNA network of 8 differentially expressed miRNAs and their target genes related to detoxification metabolism in *S. frugiperda*. Red triangles represent upregulated miRNAs while blue triangles represent downregulated miRNAs. Other circles represent different target genes related to detoxification metabolism.

To better understand the interactions between miRNAs and their potential target genes, eight of the most significantly differentially expressed miRNAs and detoxification metabolism-related target mRNAs were selected to construct a miRNA–mRNA network (Figure 10). The eight miRNAs included the four most upregulated miRNAs in larvae (sfr-miR-14-5p, sfr-miR-6094-5p, sfr-miR-6094-3p, and sfr-miR-34-5p) and the four most downregulated miRNAs (sfr-miR-2765-5p, sfr-miR-279b-3p, sfr-miR-277-3p, and sfr-miR-307-3p). A total of 82 target genes were identified, targeting 10 gene families involved in detoxification metabolism. These included twenty-nine genes in the P450 gene family, twenty-four in the ABC transporters gene family, thirteen in the GSTs gene family, four in the CarEs gene family, nine in the Acetylcholine gene family, twenty-three in the Sodium channel gene family, seven in the Ryanodine gene family, eight in the Cadherin gene family, seven in the Alkaline phosphatase gene family, and eleven in the Aminopeptidase gene family. These results suggest that detoxification metabolism-related pathways play a critical role in insecticide resistance in *S. frugiperda*.

## 4. Discussion

In recent years, increasing numbers of miRNAs have been discovered in various eukaryotic organisms [57]. In insects, miRNAs regulate gene expression at the post-transcriptional level by either repressing translation or degrading mRNA, with their functions mainly focused on various physiological processes in insects [58,59,60,61]. The larval stage is the primary period during which *S. frugiperda* infests crops like corn [62]. It is also a crucial period for rapid growth and preparation for pupation. Studying miRNAs preferentially expressed in the larvae of the fall armyworm can aid in identifying miRNAs involved in regulating growth and development, as well as analyzing detoxification-related target genes, providing new insights and methods for effectively controlling outbreaks and damage caused by this pest.

Chemical control of *S. frugiperda* has a long history, so a variety of resistance mechanisms have emerged [63]. In the United States and Brazil, chemical control of *S. frugiperda* is mainly used before planting Bt transgenic maize [39]. The metabolic capacity of carbaryl -resistant strains is five times higher than that of sensitive strains, and the resistance is mainly caused by oxidative metabolism such as P450 hydroxylation and epoxidation [64]. The activities of various detoxification metabolic enzymes such as multifunctional oxidase (MFO), GSTs, and esterase (ESTs) of laboratory-resistant strains are significantly higher than those of sensitive strains [37]. The sensitivity of acetylcholinesterase (AChE), a molecular target of carbamate and organophosphorus insecticides, to menafarb is also significantly reduced [65]. All these indicate that the resistance of *S. frugiperda* in the field population is caused by several mechanisms, so the study of insecticide resistance is particularly important in chemical control.

This study also provides comprehensive insights into the sRNA landscape of *S. frugiperda* across its developmental stages, with a particular focus on identifying and predicting differentially expressed miRNAs associated with detoxification pathways in larvae. The results revealed critical patterns and potential regulatory roles of miRNAs, which could be crucial for understanding and managing this pest’s resistance to insecticides.

Our analysis identified a substantial number of known and novel miRNAs across the developmental stages of *S. frugiperda*, including 135, 149, 168, and 164 known miRNAs in eggs, larvae, pupae, and adults, respectively. Additionally, we discovered 147, 83, 122, and 138 novel miRNAs in these stages. These findings are consistent with previous studies that have identified numerous miRNAs in various insect species [31,43,66,67,68,69], underscoring the conserved nature of miRNA-mediated regulation in insect development and physiology.

Notably, miR-2766-3p, miR-279a-3p, and miR-10-5p were among the most abundantly expressed miRNAs in *S. frugiperda*, with significant expression in the larval stage. These miRNAs have been implicated in crucial biological processes such as development, stress response, and resistance mechanisms in other insect species. For instance, miR-2766 regulates tyrosine hydroxylase in *H. armigera*, influencing larval–pupal metamorphosis [70]. Similarly, miR-279a-3p modulates CYP325BB1 expression, affecting insecticide resistance in mosquitoes [71]. In addition, miR-10 is also the most abundantly expressed miRNA in larvae of *H. armigera* when parasitized by the wasp *Diadegma semiclausum* [72].

The differential expression analysis highlighted eight miRNAs with significant changes in expression levels in *S. frugiperda* larvae compared to other developmental stages. These include upregulated miRNAs such as sfr-miR-6094-3p, sfr-miR-6094-5p, and sfr-miR-34-5p, and downregulated miRNAs such as sfr-miR-2765-5p and sfr-miR-279b-3p. The high expression of sfr-miR-34-5p in larvae and its decrease in eggs and pupae suggest its pivotal role in larval development. This pattern aligns with previous findings in other insects, where miRNAs were also highly expressed in larvae compared to other stages [73,74,75,76,77,78].

The KEGG and GO enrichment analyses of target genes predicted for differentially expressed miRNAs revealed their involvement in critical biological pathways, including detoxification metabolism and G protein-coupled receptor (GPCR) signaling. GPCR signaling has been linked to insecticide resistance in various insects, including *S. frugiperda* [79]. Our study identified 82 potential detoxification-related target genes regulated by differentially expressed miRNAs, including genes from the P450, GST, and ABC transporter families. These gene families are well-known for their roles in insecticide detoxification and resistance [80].

Previous studies have demonstrated that miRNAs can modulate insecticide sensitivity by targeting detoxification genes. For instance, miR-2b-3p and miR-14-5p in *P. xylostella* larvae were shown to influence the expression of CYP9F2 and CYP307a1, affecting the larval detoxification capacity [81]. Similarly, regulating novel_miR-1517 expression in *Bemisia tabaci* affected its sensitivity to imidacloprid by modulating CYP6CM1 expression [82]. The chlorpyrifos- and cypermethrin-resistant nature of *S. frugiperda* strains showed that the A201S, G227A, and F290V point mutations of AChE are resistant to chlorpyrifos [39]. The T929I, L932F, and L1014F point mutations in sodium ion channels lead to resistance to cyhalothrin. The increased expressions of P450, GSTs, and ESTs are involved in resistance to chlorpyrifos and cypermethrin [83]. Our findings suggest that miRNAs in *S. frugiperda* may similarly regulate detoxification genes, contributing to its resistance to various insecticides.

The findings of this study have significant implications for understanding the molecular mechanisms underlying insecticide resistance in *S. frugiperda*. The identified miRNAs and their target genes involved in detoxification pathways offer potential targets for developing novel pest management strategies. By manipulating specific miRNAs or their targets, it might be possible to enhance the efficacy of insecticides or develop new control methods that circumvent existing resistance mechanisms. Future research should focus on functional validation of these miRNAs and their targets using techniques such as miRNA mimics and inhibitors, gene knockout or knockdown experiments, and biochemical assays to confirm their roles in detoxification and resistance. Additionally, exploring the environmental and physiological factors that influence miRNA expression and activity will further elucidate their regulatory networks in *S. frugiperda*.

## 5. Conclusions

In summary, eight miRNAs showed differential expression in *S. frugiperda* larvae compared to the other three stages. A total of 82 target genes were identified, targeting 10 gene families involved in detoxification metabolism. These results provide a theoretical basis for further exploring the mechanism of miRNAs regulating detoxification metabolism genes.

## Figures and Tables

**Table 1:** Primers used for the qRT-PCR.

Primer	Sequence (from 5′ End to 3′ End)
miR-6094-5P-RT	GTCGTATCCAGTGCAGGGTCCGAGGTAT TCGCACTGGATACGACAGGTAC
miR-6094-5P-F	TCAGCGGTGGCCTGGG
miR-6094-3P-RT	GTCGTATCCAGTGCAGGGTCCGAGGTATTCGCACTGGATACGACAGGATC
miR-6094-3P-F	CGCGTATTCGAGACCTCTGCT
miR-10505-3p-RT	GTCGTATCCAGTGCAGGGTCCGAGGTATTCGCACTGGATACGACGAGCCA
miR-10505-3p-F	CGCGCGTAGGGTTAGAAACT
miR-14-5p-RT	GTCGTATCCAGTGCAGGGTCCGAGGTATTCGCACTGGATACGACTCGAGT
miR-14-5p-F	GCGGGGGGAGGAATTG
miR-2765-5p-RT	GTCGTATCCAGTGCAGGGTCCGAGGTATTCGCACTGGATACGACGCCAAC
miR-2765-5p-F	CGCGCGTGGTAACTCCACCACC
miR-277-3p-RT	GTCGTATCCAGTGCAGGGTCCGAGGTATTCGCACTGGATACGACTGTCGT
miR-277-3p-F	CGCGCGTAAATGCACTATCTGGT
miR-307-3p-RT	GTCGTATCCAGTGCAGGGTCCGAGGTATTCGCACTGGATACGACGCTCAC
miR-307-3p-F	CGCGCGTCACAACCTCCTTGA
miR-34-5p-RT	GTCGTATCCAGTGCAGGGTCCGAGGTATTCGCACTGGATACGACACAACC
miR-34-5p-F	CGCGCGTGGCAGTGTGGTTAGCT
miR-R	AGTGCAGGGTCCGAGGTATT
Sf-actin(nei)-F	CGAGAAGATGACCCAGAT
Sf-actin(nei)-R	GATAGCACAGCCTGGATA
probe	FAM-CGCACTGGATACGAC-MGB

**Table 2:** Summary of sequencing reads.

Sample	Flag	Raw Reads	Average Raw Reads	Clean Reads	Average Clean Reads
Egg-1	E01	23,489,347	20,128,612	20,154,610	17,231,905
Egg-2	E02	24,772,159	20,128,612	20,586,799	17,231,905
Egg-3	E03	12,124,331	20,128,612	10,954,307	17,231,905
Larva-1	L01	10,837,072	16,315,920	1,502,965	3,731,980
Larva-2	L02	17,172,714	16,315,920	2,494,144	3,731,980
Larva-3	L03	20,937,973	16,315,920	7,198,831	3,731,980
Pupa-1	P01	20,479,951	23,520,239	14,980,746	17,141,575
Pupa-2	P02	25,798,866	23,520,239	19,742,017	17,141,575
Pupa-3	P03	24,281,901	23,520,239	16,701,963	17,141,575
Adult-1	A01	23,403,358	17,593,809	13,367,017	12,107,130
Adult-2	A02	12,435,180	17,593,809	10,254,039	12,107,130
Adult-3	A03	16,942,888	17,593,809	12,700,333	12,107,130

**Table 3:** The number of differentially expressed miRNAs in different stages of *S. frugiperda*.

Control	Case	Upregulated Genes	Downregulated Genes	Total DEGs
Egg	Larva	32	22	54
Adult	Larva	9	9	18
Pupa	Larva	6	9	15

## Data Availability

The original data presented in the study are openly available in FigShare at https://doi.org/10.6084/m9.figshare.26181866.v1.

## Supplementary Materials

The following supporting information can be downloaded at: https://www.mdpi.com/article/10.3390/genes15081021/s1, Table S1: Annotation of sRNAs in *S. frugiperda.* Table S2: Ten of the most highly expressed miRNAs in *S. frugiperda.* Numbers indicate absolute read counts.
