# Supplementary material for: Identification and Prediction of Differentially Expressed MicroRNAs Associated with Detoxification Pathways in Larvae of Spodoptera frugiperda
Source: Genes (Basel). 2024 Aug 3;15(8):1021. doi: 10.3390/genes15081021 (PMC11353827; doi:10.3390/genes15081021)
Supplement: Supplementary file 1 [file genes-15-01021-s001.zip › Supplementary file.pdf]

Identification and prediction of differentially expressed microRNAs associated with detoxification pathways in larvae of *Spodoptera frugiperda*

Yan-Ping Wang [1], Xing-Yu Chen [2], De-Qiang Pu [1], Chun-Yan Yi [1], Chang-Hua Liu [1], Cui-Cui Zhang [1], Zhen-Zhen Wei [1], Jing-Wei Guo [1], Wen-Juan Yu [1], Song Chen [1] and Hong-Ling Liu [1,*]

[1] Key Laboratory of Integrated Pest Management of Southwest Crops, Institute of Plant Protection, Sichuan Academy of Agricultural Sciences, Chengdu 610066, China; singular2023@outlook.com (Y.-P.W.); pdqpudeqiang@163.com (D.-Q.P.); yichunyan2023@outlook.com (C.-Y.Y.); liuchanghua2023@outlook.com (C.-H.L.); zhangcuicui2023@outlook.com (C.-C.Z.); zhenzhen-wei@hotmail.com (Z.-Z.W.); jingwei.guo66@hotmail.com (J.-W.G.); wjyu0906@163.com (W.-J.Y.); chensong523@126.com (S.C.);

[2] Science and Technology Security Center, Sichuan Academy of Agricultural Sciences, Chengdu 610066, China; chenxingyu2023@outlook.com (X.-Y.C.);

* Correspondence: liuhongling@ippsaas.org.cn (H.-L.L.)

Table S1. Annotation of sRNAs in *S. frugiperda*.

Type	known miRNA %	rRNA %	tRNA %	snRNA %	snoRNA %	exon %	intron %	novo miRNA %	unknown %
E01	0.98	4.36	0.15	0.00	0.00	2.32	28.13	4.03	60.02
E03	1.47	5.09	0.22	0.00	0.00	3.16	36.29	3.48	50.29
E02	1.19	6.60	0.13	0.00	0.00	2.70	27.16	4.34	57.87
A02	7.62	9.52	0.61	0.01	0.01	31.31	12.39	0.61	37.93
A03	5.90	11.27	0.15	0.00	0.01	6.65	18.42	0.74	56.86
L01	9.00	12.62	0.12	0.00	0.01	7.64	15.58	4.40	50.62
P02	2.19	13.61	0.07	0.00	0.00	6.01	6.05	1.59	70.48
P01	1.71	14.21	0.05	0.00	0.00	4.94	4.10	2.37	72.62
L03	5.72	16.95	0.11	0.00	0.01	9.14	10.20	3.55	54.32
A01	7.32	25.07	0.49	0.03	0.03	27.89	10.61	0.49	28.08
L02	3.77	25.94	0.18	0.01	0.02	7.18	5.93	1.15	55.83
P03	4.22	37.82	0.19	0.00	0.00	4.98	6.11	1.12	45.57

Table S2. Ten of the most highly expressed miRNAs in *S. frugiperda.* Numbers indicate absolute read counts.

miRNA ID	Egg	Larva	Pupa	Adult	Expression level
sfr-miR-2766-3p	112671.0667	621747.0367	324379.8167	485039.8333	high
sfr-miR-279a-3p	363417.5333	93431.7	236742.8067	237240.3067	high
sfr-miR-10-5p	161227.71	87721.67	67200.60667	22611.33333	high
sfr-miR-11-3p	120132.3267	21610.32667	46551.99667	17348.53667	high
sfr-miR-10482-5p	5104.77	3637.946667	13614.78333	8692.153333	high
sfr-miR-7-5p	81866.93333	3959.19	9430.4	1827.883333	high

sfr-miR-2755-3p	3928.06	5260.47	12486.94	5337.056667	high
sfr-miR-2766-5p	1644.913333	6002.296667	2883.713333	5609.33	high
sfr-miR-10-3p	30308.53	14710.62333	10460.9	4852.546667	high
sfr-miR-279b-3p	5499.813333	1182.26	4488.296667	7266.773333	high
